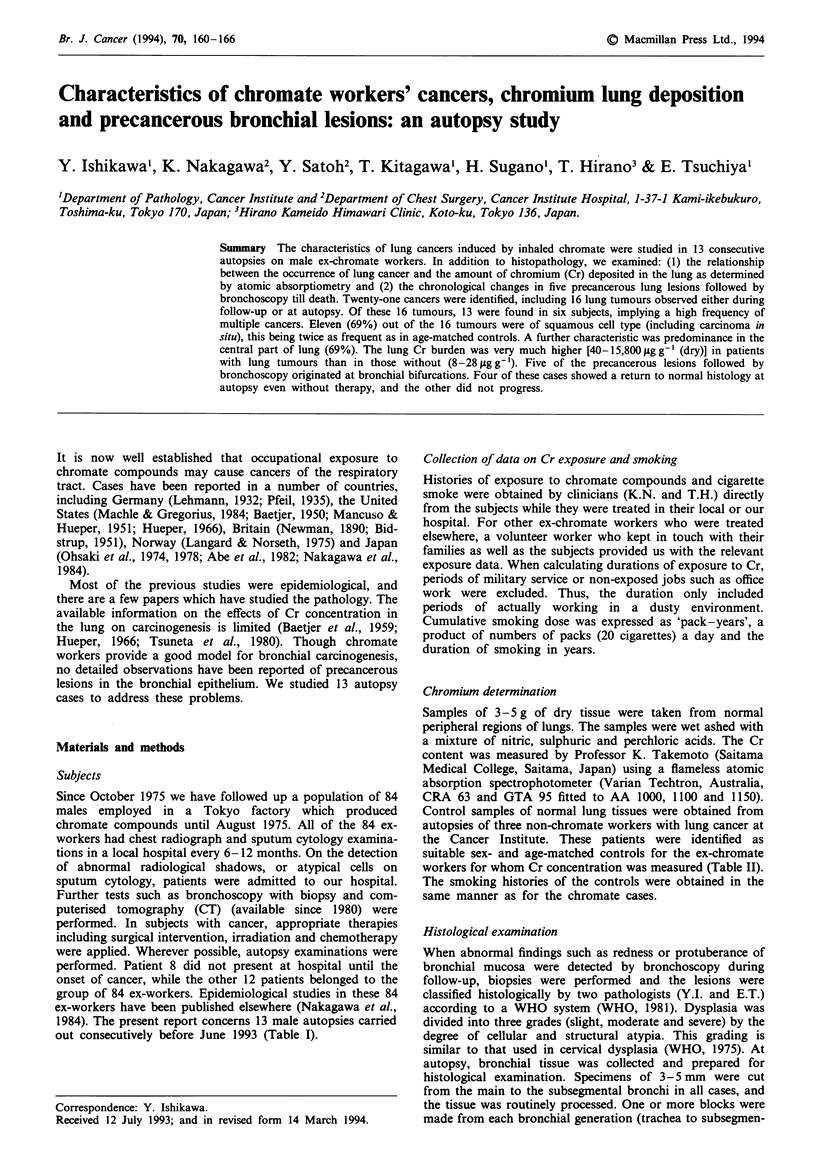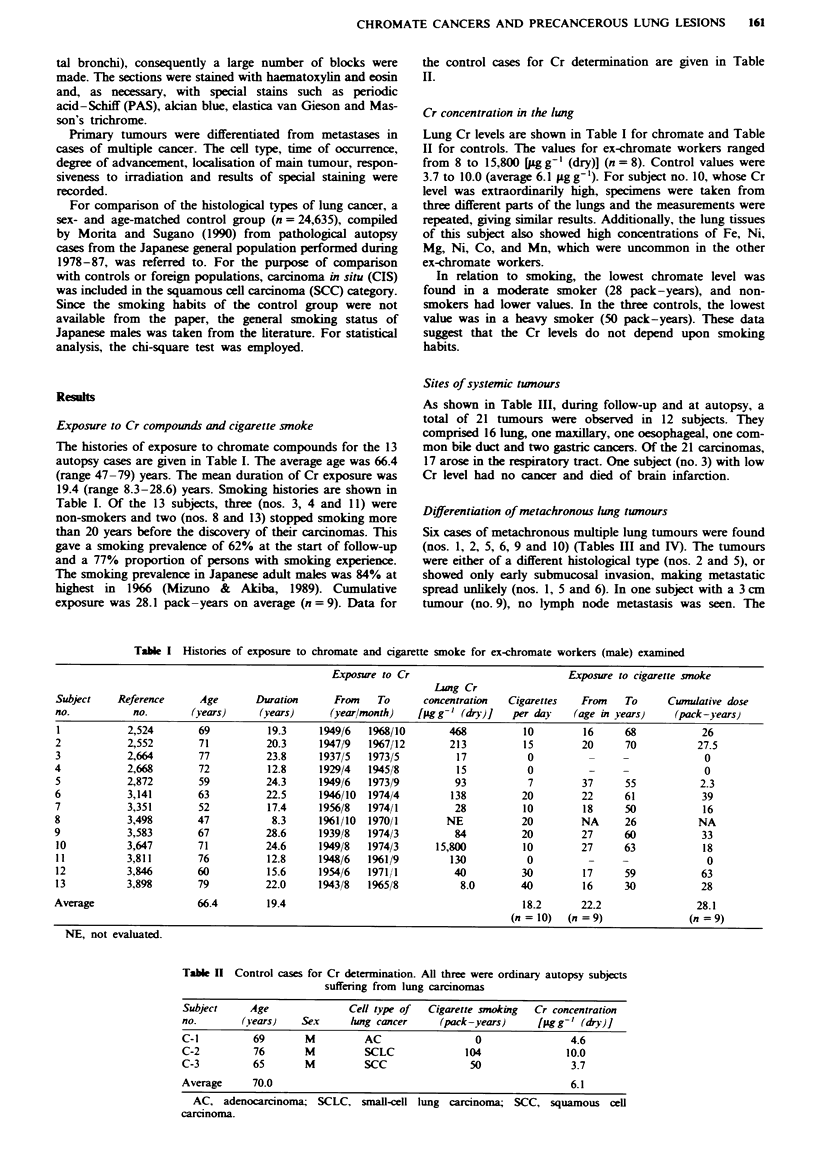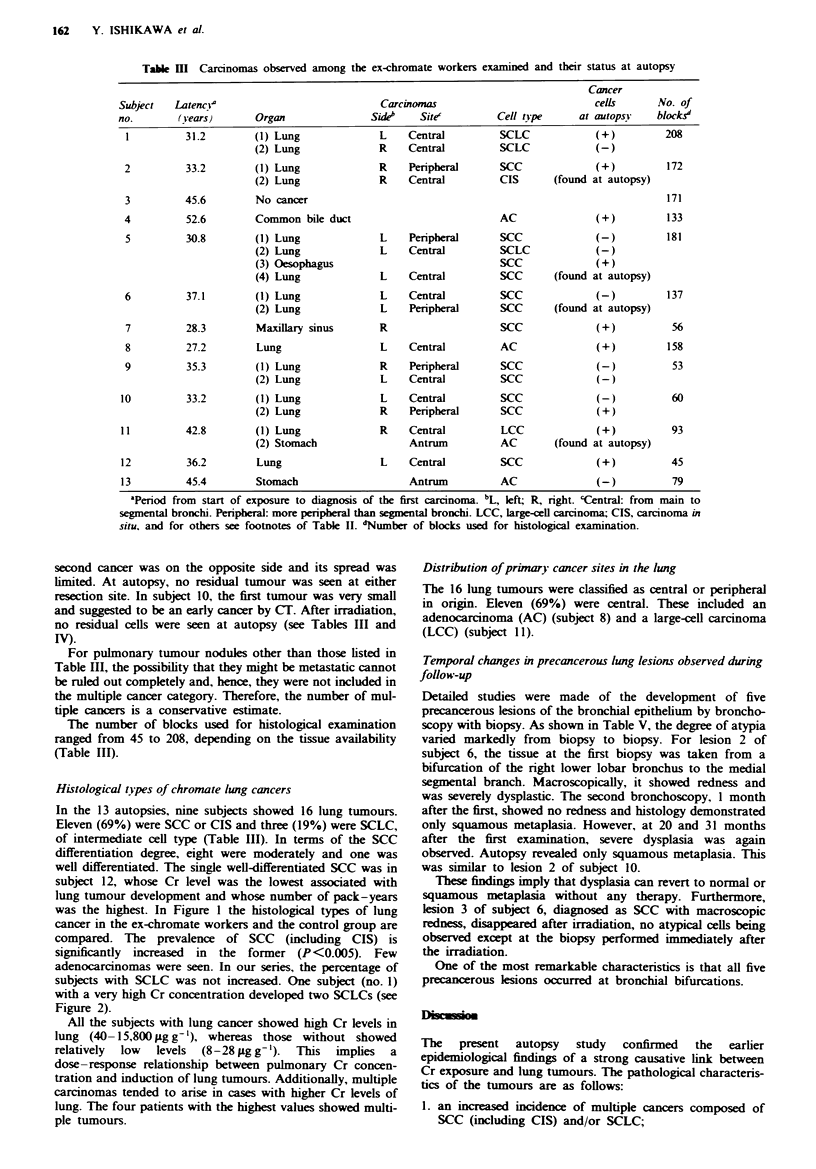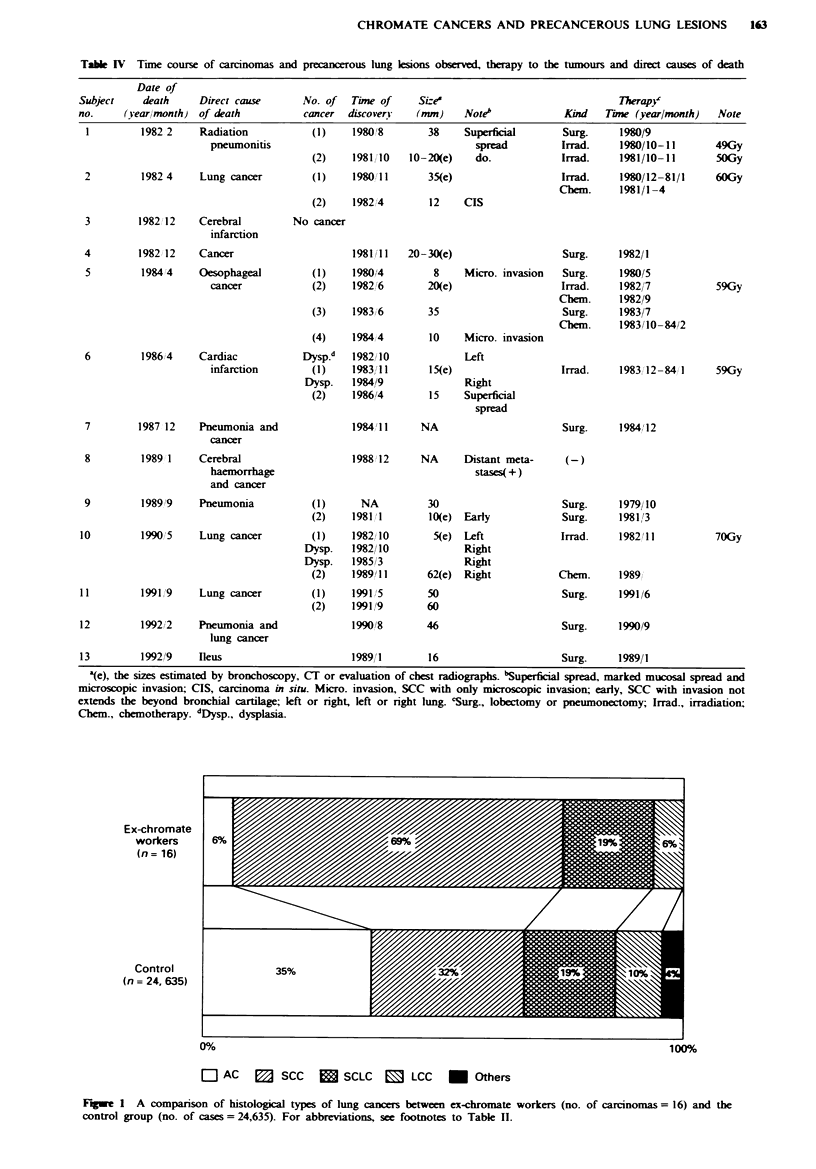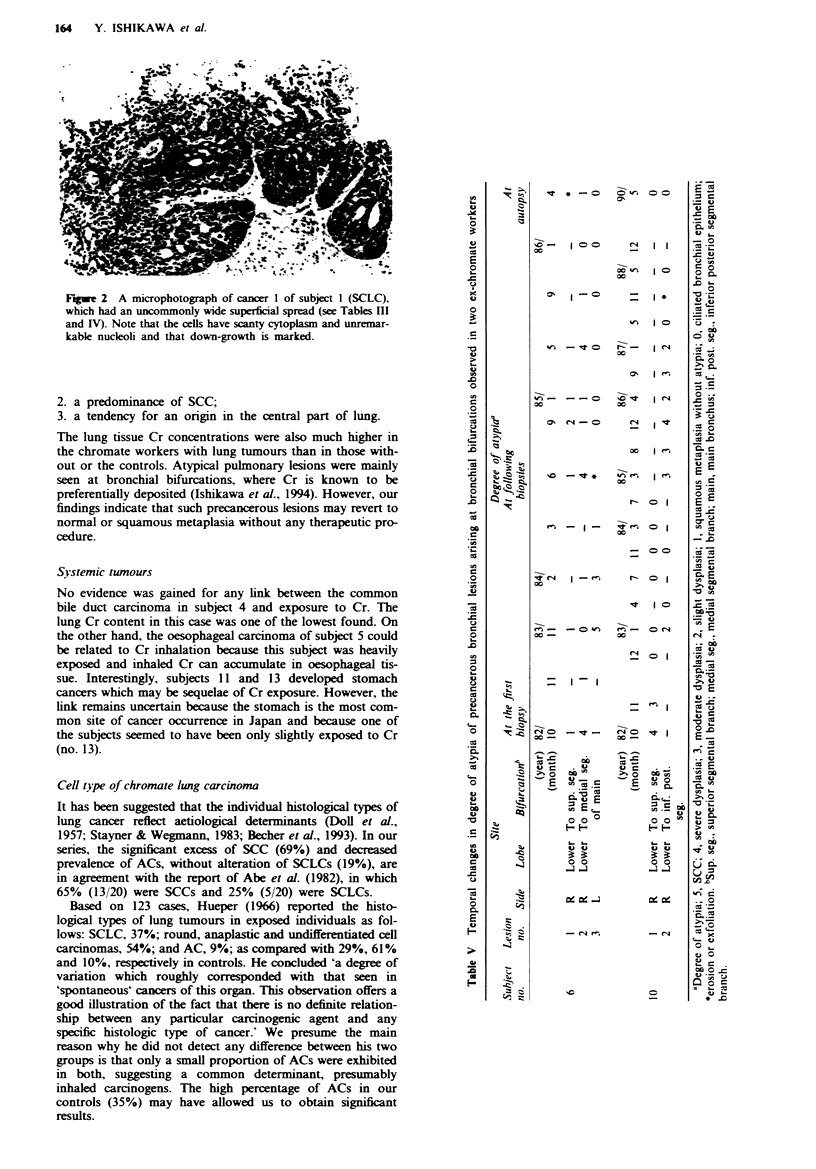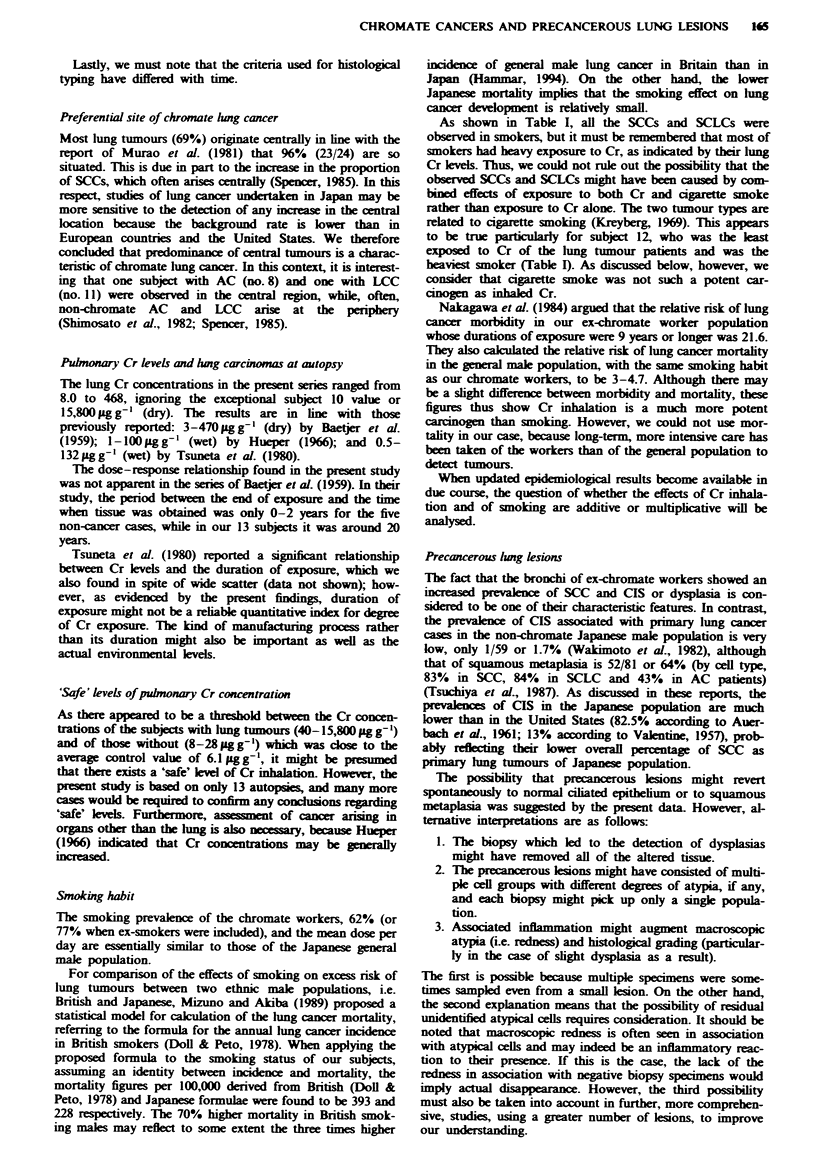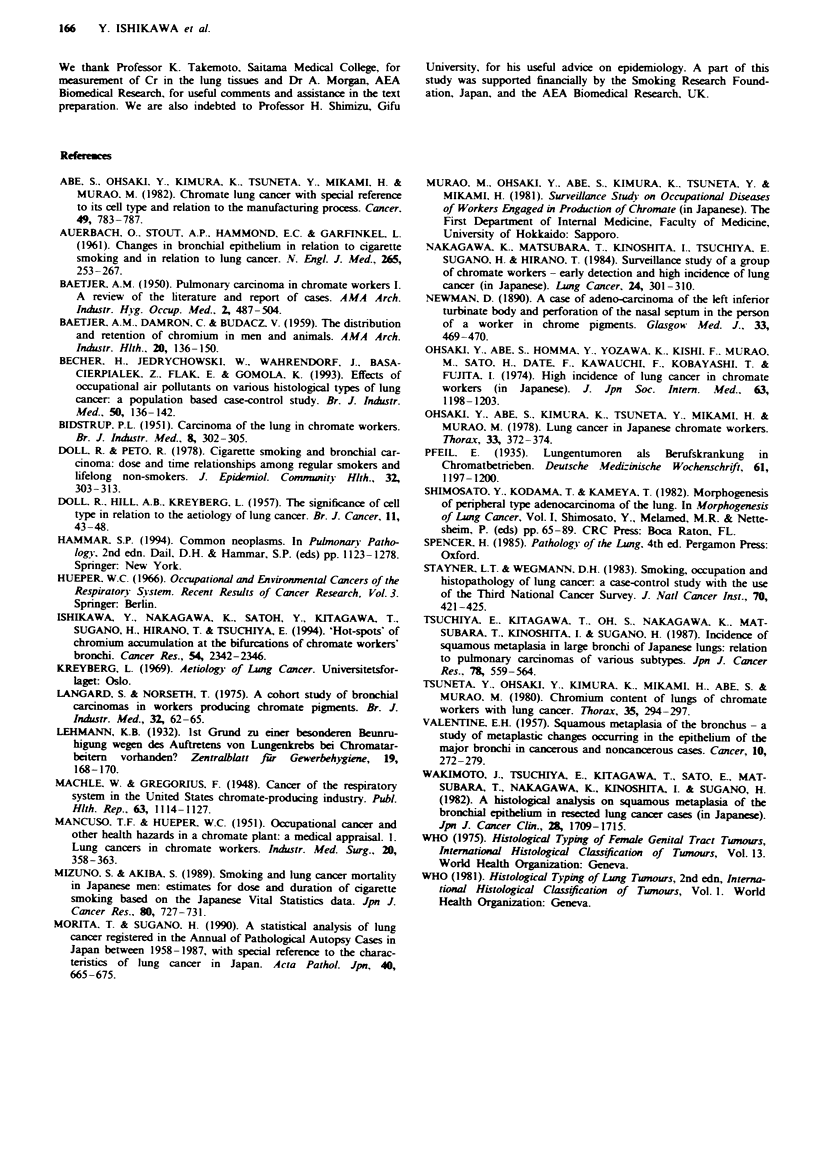# Characteristics of chromate workers' cancers, chromium lung deposition and precancerous bronchial lesions: an autopsy study.

**DOI:** 10.1038/bjc.1994.268

**Published:** 1994-07

**Authors:** Y. Ishikawa, K. Nakagawa, Y. Satoh, T. Kitagawa, H. Sugano, T. Hirano, E. Tsuchiya

**Affiliations:** Department of Pathology, Cancer Institute, Tokyo, Japan.

## Abstract

**Images:**


 © Macmillan Press Ltd., 1994

### Characteristics of chromate workers' cancers, chromium lung deposition and precancerous bronchial lesions: an autopsy study

Y. Ishikawa[1], K. Nakagawa[2], Y. Satoh[2], T. Kitagawa[1], H. Sugano[1], T. Hirano[3] & E. Tsuchiya[1]

[1]Department of Pathology, Cancer Institute and [2]Department of Chest Surgery, Cancer Institute Hospital, 1-37-1 Kami-ikebukuro, Toshima-ku, Tokyo 170, Japan; [3]Hirano Kameido Himawari Clinic, Koto-ku, Tokyo 136, Japan.

**Summary** The characteristics of lung cancers induced by inhaled chromate were studied in 13 consecutive autopsies on male ex-chromate workers. In addition to histopathology, we examined: (1) the relationship between the occurrence of lung cancer and the amount of chromium (Cr) deposited in the lung as determined by atomic absorptiometry and (2) the chronological changes in five precancerous lung lesions followed by bronchoscopy till death. Twenty-one cancers were identified, including 16 lung tumours observed either during follow-up or at autopsy. Of these 16 tumours, 13 were found in six subjects, implying a high frequency of multiple cancers. Eleven (69%) out of the 16 tumours were of squamous cell type (including carcinoma *in situ*), this being twice as frequent as in age-matched controls. A further characteristic was predominance in the central part of lung (69%). The lung Cr burden was very much higher [40-15,800 $\mu g\ g^{-1}$ (dry)] in patients with lung tumours than in those without (8-28 $\mu g\ g^{-1}$). Five of the precancerous lesions followed by bronchoscopy originated at bronchial bifurcations. Four of these cases showed a return to normal histology at autopsy even without therapy, and the other did not progress.

It is now well established that occupational exposure to chromate compounds may cause cancers of the respiratory tract. Cases have been reported in a number of countries, including Germany (Lehmann, 1932; Pfeil, 1935), the United States (Machle & Gregorius, 1984; Baetjer, 1950; Mancuso & Hueper, 1951; Hueper, 1966), Britain (Newman, 1890; Bidstrup, 1951), Norway (Langard & Norseth, 1975) and Japan (Ohsaki *et al.*, 1974, 1978; Abe *et al.*, 1982; Nakagawa *et al.*, 1984).

Most of the previous studies were epidemiological, and there are a few papers which have studied the pathology. The available information on the effects of Cr concentration in the lung on carcinogenesis is limited (Baetjer *et al.*, 1959; Hueper, 1966; Tsuneta *et al.*, 1980). Though chromate workers provide a good model for bronchial carcinogenesis, no detailed observations have been reported of precancerous lesions in the bronchial epithelium. We studied 13 autopsy cases to address these problems.

### Materials and methods

### *Subjects*

Since October 1975 we have followed up a population of 84 males employed in a Tokyo factory which produced chromate compounds until August 1975. All of the 84 ex-workers had chest radiograph and sputum cytology examinations in a local hospital every 6-12 months. On the detection of abnormal radiological shadows, or atypical cells on sputum cytology, patients were admitted to our hospital. Further tests such as bronchoscopy with biopsy and computerised tomography (CT) (available since 1980) were performed. In subjects with cancer, appropriate therapies including surgical intervention, irradiation and chemotherapy were applied. Wherever possible, autopsy examinations were performed. Patient 8 did not present at hospital until the onset of cancer, while the other 12 patients belonged to the group of 84 ex-workers. Epidemiological studies in these 84 ex-workers have been published elsewhere (Nakagawa *et al.*, 1984). The present report concerns 13 male autopsies carried out consecutively before June 1993 (Table I).

Correspondence: Y. Ishikawa.
Received 12 July 1993; and in revised form 14 March 1994.

### *Collection of data on Cr exposure and smoking*

Histories of exposure to chromate compounds and cigarette smoke were obtained by clinicians (K.N. and T.H.) directly from the subjects while they were treated in their local or our hospital. For other ex-chromate workers who were treated elsewhere, a volunteer worker who kept in touch with their families as well as the subjects provided us with the relevant exposure data. When calculating durations of exposure to Cr, periods of military service or non-exposed jobs such as office work were excluded. Thus, the duration only included periods of actually working in a dusty environment. Cumulative smoking dose was expressed as 'pack-years', a product of numbers of packs (20 cigarettes) a day and the duration of smoking in years.

### *Chromium determination*

Samples of 3-5 g of dry tissue were taken from normal peripheral regions of lungs. The samples were wet ashed with a mixture of nitric, sulphuric and perchloric acids. The Cr content was measured by Professor K. Takemoto (Saitama Medical College, Saitama, Japan) using a flameless atomic absorption spectrophotometer (Varian Techtron, Australia, CRA 63 and GTA 95 fitted to AA 1000, 1100 and 1150). Control samples of normal lung tissues were obtained from autopsies of three non-chromate workers with lung cancer at the Cancer Institute. These patients were identified as suitable sex- and age-matched controls for the ex-chromate workers for whom Cr concentration was measured (Table II). The smoking histories of the controls were obtained in the same manner as for the chromate cases.

### *Histological examination*

When abnormal findings such as redness or protuberance of bronchial mucosa were detected by bronchoscopy during follow-up, biopsies were performed and the lesions were classified histologically by two pathologists (Y.I. and E.T.) according to a WHO system (WHO, 1981). Dysplasia was divided into three grades (slight, moderate and severe) by the degree of cellular and structural atypia. This grading is similar to that used in cervical dysplasia (WHO, 1975). At autopsy, bronchial tissue was collected and prepared for histological examination. Specimens of 3-5 mm were cut from the main to the subsegmental bronchi in all cases, and the tissue was routinely processed. One or more blocks were made from each bronchial generation (trachea to subsegmental bronchi), consequently a large number of blocks were made. The sections were stained with haematoxylin and eosin and, as necessary, with special stains such as periodic acid–Schiff (PAS), alcian blue, elastica van Gieson and Masson's trichrome.

Primary tumours were differentiated from metastases in cases of multiple cancer. The cell type, time of occurrence, degree of advancement, localisation of main tumour, responsiveness to irradiation and results of special staining were recorded.

For comparison of the histological types of lung cancer, a sex- and age-matched control group ($n = 24,635$), compiled by Morita and Sugano (1990) from pathological autopsy cases from the Japanese general population performed during 1978–87, was referred to. For the purpose of comparison with controls or foreign populations, carcinoma *in situ* (CIS) was included in the squamous cell carcinoma (SCC) category. Since the smoking habits of the control group were not available from the paper, the general smoking status of Japanese males was taken from the literature. For statistical analysis, the chi-square test was employed.

### Results

### *Exposure to Cr compounds and cigarette smoke*

The histories of exposure to chromate compounds for the 13 autopsy cases are given in Table I. The average age was 66.4 (range 47–79) years. The mean duration of Cr exposure was 19.4 (range 8.3–28.6) years. Smoking histories are shown in Table I. Of the 13 subjects, three (nos. 3, 4 and 11) were non-smokers and two (nos. 8 and 13) stopped smoking more than 20 years before the discovery of their carcinomas. This gave a smoking prevalence of 62% at the start of follow-up and a 77% proportion of persons with smoking experience. The smoking prevalence in Japanese adult males was 84% at highest in 1966 (Mizuno & Akiba, 1989). Cumulative exposure was 28.1 pack–years on average ($n = 9$). Data for the control cases for Cr determination are given in Table II.

### *Cr concentration in the lung*

Lung Cr levels are shown in Table I for chromate and Table II for controls. The values for ex-chromate workers ranged from 8 to 15,800 [$\mu g\ g^{-1}$ (dry)] ($n = 8$). Control values were 3.7 to 10.0 (average 6.1 $\mu g\ g^{-1}$). For subject no. 10, whose Cr level was extraordinarily high, specimens were taken from three different parts of the lungs and the measurements were repeated, giving similar results. Additionally, the lung tissues of this subject also showed high concentrations of Fe, Ni, Mg, Ni, Co, and Mn, which were uncommon in the other ex-chromate workers.

In relation to smoking, the lowest chromate level was found in a moderate smoker (28 pack–years), and non-smokers had lower values. In the three controls, the lowest value was in a heavy smoker (50 pack–years). These data suggest that the Cr levels do not depend upon smoking habits.

### *Sites of systemic tumours*

As shown in Table III, during follow-up and at autopsy, a total of 21 tumours were observed in 12 subjects. They comprised 16 lung, one maxillary, one oesophageal, one common bile duct and two gastric cancers. Of the 21 carcinomas, 17 arose in the respiratory tract. One subject (no. 3) with low Cr level had no cancer and died of brain infarction.

### *Differentiation of metachronous lung tumours*

Six cases of metachronous multiple lung tumours were found (nos. 1, 2, 5, 6, 9 and 10) (Tables III and IV). The tumours were either of a different histological type (nos. 2 and 5), or showed only early submucosal invasion, making metastatic spread unlikely (nos. 1, 5 and 6). In one subject with a 3 cm tumour (no. 9), no lymph node metastasis was seen. The

**Table I** Histories of exposure to chromate and cigarette smoke for ex-chromate workers (male) examined

| | | | Exposure to Cr | | | | Exposure to cigarette smoke | | | |
|---|---|---|---|---|---|---|---|---|---|---|
| *Subject no.* | *Reference no.* | *Age (years)* | *Duration (years)* | *From (year/month)* | *To (year/month)* | *Lung Cr concentration [$\mu g\ g^{-1}$ (dry)]* | *Cigarettes per day* | *From (age in years)* | *To (age in years)* | *Cumulative dose (pack–years)* |
| 1 | 2,524 | 69 | 19.3 | 1949/6 | 1968/10 | 468 | 10 | 16 | 68 | 26 |
| 2 | 2,552 | 71 | 20.3 | 1947/9 | 1967/12 | 213 | 15 | 20 | 70 | 27.5 |
| 3 | 2,664 | 77 | 23.8 | 1937/5 | 1973/5 | 17 | 0 | – | – | 0 |
| 4 | 2,668 | 72 | 12.8 | 1929/4 | 1945/8 | 15 | 0 | – | – | 0 |
| 5 | 2,872 | 59 | 24.3 | 1949/6 | 1973/9 | 93 | 7 | 37 | 55 | 2.3 |
| 6 | 3,141 | 63 | 22.5 | 1946/10 | 1974/4 | 138 | 20 | 22 | 61 | 39 |
| 7 | 3,351 | 52 | 17.4 | 1956/8 | 1974/1 | 28 | 10 | 18 | 50 | 16 |
| 8 | 3,498 | 47 | 8.3 | 1961/10 | 1970/1 | NE | 20 | NA | 26 | NA |
| 9 | 3,583 | 67 | 28.6 | 1939/8 | 1974/3 | 84 | 20 | 27 | 60 | 33 |
| 10 | 3,647 | 71 | 24.6 | 1949/8 | 1974/3 | 15,800 | 10 | 27 | 63 | 18 |
| 11 | 3,811 | 76 | 12.8 | 1948/6 | 1961/9 | 130 | 0 | – | – | 0 |
| 12 | 3,846 | 60 | 15.6 | 1954/6 | 1971/1 | 40 | 30 | 17 | 59 | 63 |
| 13 | 3,898 | 79 | 22.0 | 1943/8 | 1965/8 | 8.0 | 40 | 16 | 30 | 28 |
| Average | | 66.4 | 19.4 | | | | 18.2 ($n = 10$) | 22.2 ($n = 9$) | | 28.1 ($n = 9$) |

NE, not evaluated.

**Table II** Control cases for Cr determination. All three were ordinary autopsy subjects suffering from lung carcinomas

| *Subject no.* | *Age (years)* | *Sex* | *Cell type of lung cancer* | *Cigarette smoking (pack–years)* | *Cr concentration [$\mu g\ g^{-1}$ (dry)]* |
|---|---|---|---|---|---|
| C-1 | 69 | M | AC | 0 | 4.6 |
| C-2 | 76 | M | SCLC | 104 | 10.0 |
| C-3 | 65 | M | SCC | 50 | 3.7 |
| Average | 70.0 | | | | 6.1 |

AC, adenocarcinoma; SCLC, small-cell lung carcinoma; SCC, squamous cell carcinoma.

**Table III** Carcinomas observed among the ex-chromate workers examined and their status at autopsy

| Subject no. | Latency[a] (years) | Organ | Carcinomas Side[b] | Site[c] | Cell type | Cancer cells at autopsy | No. of blocks[d] |
|---|---|---|---|---|---|---|---|
| 1 | 31.2 | (1) Lung | L | Central | SCLC | (+) | 208 |
| | | (2) Lung | R | Central | SCLC | (−) | |
| 2 | 33.2 | (1) Lung | R | Peripheral | SCC | (+) | 172 |
| | | (2) Lung | R | Central | CIS | (found at autopsy) | |
| 3 | 45.6 | No cancer | | | | | 171 |
| 4 | 52.6 | Common bile duct | | | AC | (+) | 133 |
| 5 | 30.8 | (1) Lung | L | Peripheral | SCC | (−) | 181 |
| | | (2) Lung | L | Central | SCLC | (−) | |
| | | (3) Oesophagus | | | SCC | (+) | |
| | | (4) Lung | L | Central | SCC | (found at autopsy) | |
| 6 | 37.1 | (1) Lung | L | Central | SCC | (−) | 137 |
| | | (2) Lung | L | Peripheral | SCC | (found at autopsy) | |
| 7 | 28.3 | Maxillary sinus | R | | SCC | (+) | 56 |
| 8 | 27.2 | Lung | L | Central | AC | (+) | 158 |
| 9 | 35.3 | (1) Lung | R | Peripheral | SCC | (−) | 53 |
| | | (2) Lung | L | Central | SCC | (−) | |
| 10 | 33.2 | (1) Lung | L | Central | SCC | (−) | 60 |
| | | (2) Lung | R | Peripheral | SCC | (+) | |
| 11 | 42.8 | (1) Lung | R | Central | LCC | (+) | 93 |
| | | (2) Stomach | | Antrum | AC | (found at autopsy) | |
| 12 | 36.2 | Lung | L | Central | SCC | (+) | 45 |
| 13 | 45.4 | Stomach | | Antrum | AC | (−) | 79 |

[a]Period from start of exposure to diagnosis of the first carcinoma. [b]L, left; R, right. [c]Central: from main to segmental bronchi. Peripheral: more peripheral than segmental bronchi. LCC, large-cell carcinoma; CIS, carcinoma *in situ*, and for others see footnotes of Table II. [d]Number of blocks used for histological examination.

second cancer was on the opposite side and its spread was limited. At autopsy, no residual tumour was seen at either resection site. In subject 10, the first tumour was very small and suggested to be an early cancer by CT. After irradiation, no residual cells were seen at autopsy (see Tables III and IV).

For pulmonary tumour nodules other than those listed in Table III, the possibility that they might be metastatic cannot be ruled out completely and, hence, they were not included in the multiple cancer category. Therefore, the number of multiple cancers is a conservative estimate.

The number of blocks used for histological examination ranged from 45 to 208, depending on the tissue availability (Table III).

### *Histological types of chromate lung cancers*

In the 13 autopsies, nine subjects showed 16 lung tumours. Eleven (69%) were SCC or CIS and three (19%) were SCLC, of intermediate cell type (Table III). In terms of the SCC differentiation degree, eight were moderately and one was well differentiated. The single well-differentiated SCC was in subject 12, whose Cr level was the lowest associated with lung tumour development and whose number of pack-years was the highest. In Figure 1 the histological types of lung cancer in the ex-chromate workers and the control group are compared. The prevalence of SCC (including CIS) is significantly increased in the former ($P<0.005$). Few adenocarcinomas were seen. In our series, the percentage of subjects with SCLC was not increased. One subject (no. 1) with a very high Cr concentration developed two SCLCs (see Figure 2).

All the subjects with lung cancer showed high Cr levels in lung (40–15,800 $\mu g\ g^{-1}$), whereas those without showed relatively low levels (8–28 $\mu g\ g^{-1}$). This implies a dose–response relationship between pulmonary Cr concentration and induction of lung tumours. Additionally, multiple carcinomas tended to arise in cases with higher Cr levels of lung. The four patients with the highest values showed multiple tumours.

### *Distribution of primary cancer sites in the lung*

The 16 lung tumours were classified as central or peripheral in origin. Eleven (69%) were central. These included an adenocarcinoma (AC) (subject 8) and a large-cell carcinoma (LCC) (subject 11).

### *Temporal changes in precancerous lung lesions observed during follow-up*

Detailed studies were made of the development of five precancerous lesions of the bronchial epithelium by bronchoscopy with biopsy. As shown in Table V, the degree of atypia varied markedly from biopsy to biopsy. For lesion 2 of subject 6, the tissue at the first biopsy was taken from a bifurcation of the right lower lobar bronchus to the medial segmental branch. Macroscopically, it showed redness and was severely dysplastic. The second bronchoscopy, 1 month after the first, showed no redness and histology demonstrated only squamous metaplasia. However, at 20 and 31 months after the first examination, severe dysplasia was again observed. Autopsy revealed only squamous metaplasia. This was similar to lesion 2 of subject 10.

These findings imply that dysplasia can revert to normal or squamous metaplasia without any therapy. Furthermore, lesion 3 of subject 6, diagnosed as SCC with macroscopic redness, disappeared after irradiation, no atypical cells being observed except at the biopsy performed immediately after the irradiation.

One of the most remarkable characteristics is that all five precancerous lesions occurred at bronchial bifurcations.

### Discussion

The present autopsy study confirmed the earlier epidemiological findings of a strong causative link between Cr exposure and lung tumours. The pathological characteristics of the tumours are as follows:

1. an increased incidence of multiple cancers composed of SCC (including CIS) and/or SCLC;

**Table IV** Time course of carcinomas and precancerous lung lesions observed, therapy to the tumours and direct causes of death

| *Subject no.* | *Date of death (year/month)* | *Direct cause of death* | *No. of cancer* | *Time of discovery* | *Size*[a] *(mm)* | *Note*[b] | *Therapy*[c] *Kind* | *Therapy*[c] *Time (year/month)* | *Note* |
|---|---|---|---|---|---|---|---|---|---|
| 1 | 1982/2 | Radiation pneumonitis | (1) | 1980/8 | 38 | Superficial spread | Surg. | 1980/9 | |
| | | | | | | | Irrad. | 1980/10–11 | 49Gy |
| | | | (2) | 1981/10 | 10–20(e) | do. | Irrad. | 1981/10–11 | 50Gy |
| 2 | 1982/4 | Lung cancer | (1) | 1980/11 | 35(e) | | Irrad. | 1980/12–81/1 | 60Gy |
| | | | | | | | Chem. | 1981/1–4 | |
| | | | (2) | 1982/4 | 12 | CIS | | | |
| 3 | 1982/12 | Cerebral infarction | No cancer | | | | | | |
| 4 | 1982/12 | Cancer | | 1981/11 | 20–30(e) | | Surg. | 1982/1 | |
| 5 | 1984/4 | Oesophageal cancer | (1) | 1980/4 | 8 | Micro. invasion | Surg. | 1980/5 | |
| | | | (2) | 1982/6 | 20(e) | | Irrad. | 1982/7 | 59Gy |
| | | | | | | | Chem. | 1982/9 | |
| | | | (3) | 1983/6 | 35 | | Surg. | 1983/7 | |
| | | | | | | | Chem. | 1983/10–84/2 | |
| | | | (4) | 1984/4 | 10 | Micro. invasion | | | |
| 6 | 1986/4 | Cardiac infarction | Dysp.[d] | 1982/10 | | Left | | | |
| | | | (1) | 1983/11 | 15(e) | | Irrad. | 1983/12–84/1 | 59Gy |
| | | | Dysp. | 1984/9 | | Right | | | |
| | | | (2) | 1986/4 | 15 | Superficial spread | | | |
| 7 | 1987/12 | Pneumonia and cancer | | 1984/11 | NA | | Surg. | 1984/12 | |
| 8 | 1989/1 | Cerebral haemorrhage and cancer | | 1988/12 | NA | Distant metastases(+) | (−) | | |
| 9 | 1989/9 | Pneumonia | (1) | NA | 30 | | Surg. | 1979/10 | |
| | | | (2) | 1981/1 | 10(e) | Early | Surg. | 1981/3 | |
| 10 | 1990/5 | Lung cancer | (1) | 1982/10 | 5(e) | Left | Irrad. | 1982/11 | 70Gy |
| | | | Dysp. | 1982/10 | | Right | | | |
| | | | Dysp. | 1985/3 | | Right | | | |
| | | | (2) | 1989/11 | 62(e) | Right | Chem. | 1989/ | |
| 11 | 1991/9 | Lung cancer | (1) | 1991/5 | 50 | | Surg. | 1991/6 | |
| | | | (2) | 1991/9 | 60 | | | | |
| 12 | 1992/2 | Pneumonia and lung cancer | | 1990/8 | 46 | | Surg. | 1990/9 | |
| 13 | 1992/9 | Ileus | | 1989/1 | 16 | | Surg. | 1989/1 | |

[a](e), the sizes estimated by bronchoscopy, CT or evaluation of chest radiographs. [b]Superficial spread, marked mucosal spread and microscopic invasion; CIS, carcinoma *in situ*. Micro. invasion, SCC with only microscopic invasion; early, SCC with invasion not extends the beyond bronchial cartilage; left or right, left or right lung. [c]Surg., lobectomy or pneumonectomy; Irrad., irradiation; Chem., chemotherapy. [d]Dysp., dysplasia.

**Figure 1** A comparison of histological types of lung cancers between ex-chromate workers (no. of carcinomas = 16) and the control group (no. of cases = 24,635). For abbreviations, see footnotes to Table II.

**Figure 2** A microphotograph of cancer 1 of subject 1 (SCLC), which had an uncommonly wide superficial spread (see Tables III and IV). Note that the cells have scanty cytoplasm and unremarkable nucleoli and that down-growth is marked.

2. a predominance of SCC;
3. a tendency for an origin in the central part of lung.

The lung tissue Cr concentrations were also much higher in the chromate workers with lung tumours than in those without or the controls. Atypical pulmonary lesions were mainly seen at bronchial bifurcations, where Cr is known to be preferentially deposited (Ishikawa *et al.*, 1994). However, our findings indicate that such precancerous lesions may revert to normal or squamous metaplasia without any therapeutic procedure.

*Systemic tumours*

No evidence was gained for any link between the common bile duct carcinoma in subject 4 and exposure to Cr. The lung Cr content in this case was one of the lowest found. On the other hand, the oesophageal carcinoma of subject 5 could be related to Cr inhalation because this subject was heavily exposed and inhaled Cr can accumulate in oesophageal tissue. Interestingly, subjects 11 and 13 developed stomach cancers which may be sequelae of Cr exposure. However, the link remains uncertain because the stomach is the most common site of cancer occurrence in Japan and because one of the subjects seemed to have been only slightly exposed to Cr (no. 13).

*Cell type of chromate lung carcinoma*

It has been suggested that the individual histological types of lung cancer reflect aetiological determinants (Doll *et al.*, 1957; Stayner & Wegmann, 1983; Becher *et al.*, 1993). In our series, the significant excess of SCC (69%) and decreased prevalence of ACs, without alteration of SCLCs (19%), are in agreement with the report of Abe *et al.* (1982), in which 65% (13/20) were SCCs and 25% (5/20) were SCLCs.

Based on 123 cases, Hueper (1966) reported the histological types of lung tumours in exposed individuals as follows: SCLC, 37%; round, anaplastic and undifferentiated cell carcinomas, 54%; and AC, 9%; as compared with 29%, 61% and 10%, respectively in controls. He concluded 'a degree of variation which roughly corresponded with that seen in 'spontaneous' cancers of this organ. This observation offers a good illustration of the fact that there is no definite relationship between any particular carcinogenic agent and any specific histologic type of cancer.' We presume the main reason why he did not detect any difference between his two groups is that only a small proportion of ACs were exhibited in both, suggesting a common determinant, presumably inhaled carcinogens. The high percentage of ACs in our controls (35%) may have allowed us to obtain significant results.

**Table V** Temporal changes in degree of atypia of precancerous bronchial lesions arising at bronchial bifurcations observed in two ex-chromate workers

| *Subject no.* | *Lesion no.* | *Site*: *Side* | *Lobe* | *Bifurcation*[b] | Degree of atypia[a]: *At the first biopsy* (year) 82/ (month) 10 | *At following biopsies* 11 | 83/ 11 | 84/ 2 | 3 | 6 | 9 | 85/ 1 | 5 | 9 | 86/ 1 | *At autopsy* 4 |
|---|---|---|---|---|---|---|---|---|---|---|---|---|---|---|---|---|
| 6 | 1 | R | Lower | To sup. seg. | 1 | – | 1 | – | 1 | 1 | 2 | 1 | 1 | – | – | * |
| | 2 | R | Lower | To medial seg. | 4 | 1 | 0 | 1 | – | 4 | 1 | 1 | 4 | 1 | 0 | 1 |
| | 3 | L | | of main | 1 | – | 5 | 3 | 1 | * | 0 | 0 | 0 | 0 | 0 | 0 |

| *Subject no.* | *Lesion no.* | *Side* | *Lobe* | *Bifurcation*[b] | *At the first biopsy* (year) 82/ (month) 10 | 11 | 12 | 83/ 1 | 4 | 7 | 11 | 84/ 3 | 7 | 85/ 3 | 8 | 12 | 86/ 4 | 9 | 87/ 1 | 5 | 11 | 88/ 5 | 12 | *At autopsy* 90/ 5 |
|---|---|---|---|---|---|---|---|---|---|---|---|---|---|---|---|---|---|---|---|---|---|---|---|---|
| 10 | 1 | R | Lower | To sup. seg. | 4 | 3 | 0 | 0 | – | 0 | 0 | 0 | 0 | – | – | – | – | – | – | – | – | – | – | 0 |
| | 2 | R | Lower | To inf. post. seg. | – | – | – | 2 | 0 | – | 0 | – | – | 3 | 3 | 4 | 2 | 3 | 2 | 0 | * | 0 | – | 0 |

[a]Degree of atypia; 5, SCC; 4, severe dysplasia; 3, moderate dysplasia; 2, slight dysplasia; 1, squamous metaplasia without atypia; 0, ciliated bronchial epithelium; *erosion or exfoliation. [b]Sup. seg., superior segmental branch; medial seg., medial segmental branch; main, main bronchus; inf. post. seg., inferior posterior segmental branch.

Lastly, we must note that the criteria used for histological typing have differed with time.

*Preferential site of chromate lung cancer*

Most lung tumours (69%) originate centrally in line with the report of Murao *et al.* (1981) that 96% (23/24) are so situated. This is due in part to the increase in the proportion of SCCs, which often arises centrally (Spencer, 1985). In this respect, studies of lung cancer undertaken in Japan may be more sensitive to the detection of any increase in the central location because the background rate is lower than in European countries and the United States. We therefore concluded that predominance of central tumours is a characteristic of chromate lung cancer. In this context, it is interesting that one subject with AC (no. 8) and one with LCC (no. 11) were observed in the central region, while, often, non-chromate AC and LCC arise at the periphery (Shimosato *et al.*, 1982; Spencer, 1985).

*Pulmonary Cr levels and lung carcinomas at autopsy*

The lung Cr concentrations in the present series ranged from 8.0 to 468, ignoring the exceptional subject 10 value or 15,800 $\mu g\ g^{-1}$ (dry). The results are in line with those previously reported: 3–470 $\mu g\ g^{-1}$ (dry) by Baetjer *et al.* (1959); 1–100 $\mu g\ g^{-1}$ (wet) by Hueper (1966); and 0.5–132 $\mu g\ g^{-1}$ (wet) by Tsuneta *et al.* (1980).

The dose–response relationship found in the present study was not apparent in the series of Baetjer *et al.* (1959). In their study, the period between the end of exposure and the time when tissue was obtained was only 0–2 years for the five non-cancer cases, while in our 13 subjects it was around 20 years.

Tsuneta *et al.* (1980) reported a significant relationship between Cr levels and the duration of exposure, which we also found in spite of wide scatter (data not shown); however, as evidenced by the present findings, duration of exposure might not be a reliable quantitative index for degree of Cr exposure. The kind of manufacturing process rather than its duration might also be important as well as the actual environmental levels.

*'Safe' levels of pulmonary Cr concentration*

As there appeared to be a threshold between the Cr concentrations of the subjects with lung tumours (40–15,800 $\mu g\ g^{-1}$) and of those without (8–28 $\mu g\ g^{-1}$) which was close to the average control value of 6.1 $\mu g\ g^{-1}$, it might be presumed that there exists a 'safe' level of Cr inhalation. However, the present study is based on only 13 autopsies, and many more cases would be required to confirm any conclusions regarding 'safe' levels. Furthermore, assessment of cancer arising in organs other than the lung is also necessary, because Hueper (1966) indicated that Cr concentrations may be generally increased.

*Smoking habit*

The smoking prevalence of the chromate workers, 62% (or 77% when ex-smokers were included), and the mean dose per day are essentially similar to those of the Japanese general male population.

For comparison of the effects of smoking on excess risk of lung tumours between two ethnic male populations, i.e. British and Japanese, Mizuno and Akiba (1989) proposed a statistical model for calculation of the lung cancer mortality, referring to the formula for the annual lung cancer incidence in British smokers (Doll & Peto, 1978). When applying the proposed formula to the smoking status of our subjects, assuming an identity between incidence and mortality, the mortality figures per 100,000 derived from British (Doll & Peto, 1978) and Japanese formulae were found to be 393 and 228 respectively. The 70% higher mortality in British smoking males may reflect to some extent the three times higher incidence of general male lung cancer in Britain than in Japan (Hammar, 1994). On the other hand, the lower Japanese mortality implies that the smoking effect on lung cancer development is relatively small.

As shown in Table I, all the SCCs and SCLCs were observed in smokers, but it must be remembered that most of smokers had heavy exposure to Cr, as indicated by their lung Cr levels. Thus, we could not rule out the possibility that the observed SCCs and SCLCs might have been caused by combined effects of exposure to both Cr and cigarette smoke rather than exposure to Cr alone. The two tumour types are related to cigarette smoking (Kreyberg, 1969). This appears to be true particularly for subject 12, who was the least exposed to Cr of the lung tumour patients and was the heaviest smoker (Table I). As discussed below, however, we consider that cigarette smoke was not such a potent carcinogen as inhaled Cr.

Nakagawa *et al.* (1984) argued that the relative risk of lung cancer morbidity in our ex-chromate worker population whose durations of exposure were 9 years or longer was 21.6. They also calculated the relative risk of lung cancer mortality in the general male population, with the same smoking habit as our chromate workers, to be 3–4.7. Although there may be a slight difference between morbidity and mortality, these figures thus show Cr inhalation is a much more potent carcinogen than smoking. However, we could not use mortality in our case, because long-term, more intensive care has been taken of the workers than of the general population to detect tumours.

When updated epidemiological results become available in due course, the question of whether the effects of Cr inhalation and of smoking are additive or multiplicative will be analysed.

*Precancerous lung lesions*

The fact that the bronchi of ex-chromate workers showed an increased prevalence of SCC and CIS or dysplasia is considered to be one of their characteristic features. In contrast, the prevalence of CIS associated with primary lung cancer cases in the non-chromate Japanese male population is very low, only 1/59 or 1.7% (Wakimoto *et al.*, 1982), although that of squamous metaplasia is 52/81 or 64% (by cell type, 83% in SCC, 84% in SCLC and 43% in AC patients) (Tsuchiya *et al.*, 1987). As discussed in these reports, the prevalences of CIS in the Japanese population are much lower than in the United States (82.5% according to Auerbach *et al.*, 1961; 13% according to Valentine, 1957), probably reflecting their lower overall percentage of SCC as primary lung tumours of Japanese population.

The possibility that precancerous lesions might revert spontaneously to normal ciliated epithelium or to squamous metaplasia was suggested by the present data. However, alternative interpretations are as follows:

1. The biopsy which led to the detection of dysplasias might have removed all of the altered tissue.
2. The precancerous lesions might have consisted of multiple cell groups with different degrees of atypia, if any, and each biopsy might pick up only a single population.
3. Associated inflammation might augment macroscopic atypia (i.e. redness) and histological grading (particularly in the case of slight dysplasia as a result).

The first is possible because multiple specimens were sometimes sampled even from a small lesion. On the other hand, the second explanation means that the possibility of residual unidentified atypical cells requires consideration. It should be noted that macroscopic redness is often seen in association with atypical cells and may indeed be an inflammatory reaction to their presence. If this is the case, the lack of the redness in association with negative biopsy specimens would imply actual disappearance. However, the third possibility must also be taken into account in further, more comprehensive, studies, using a greater number of lesions, to improve our understanding.

We thank Professor K. Takemoto, Saitama Medical College, for measurement of Cr in the lung tissues and Dr A. Morgan, AEA Biomedical Research, for useful comments and assistance in the text preparation. We are also indebted to Professor H. Shimizu, Gifu University, for his useful advice on epidemiology. A part of this study was supported financially by the Smoking Research Foundation, Japan, and the AEA Biomedical Research, UK.

### References

ABE, S., OHSAKI, Y., KIMURA, K., TSUNETA, Y., MIKAMI, H. & MURAO, M. (1982). Chromate lung cancer with special reference to its cell type and relation to the manufacturing process. *Cancer*, **49**, 783-787.

AUERBACH, O., STOUT, A.P., HAMMOND, E.C. & GARFINKEL, L. (1961). Changes in bronchial epithelium in relation to cigarette smoking and in relation to lung cancer. *N. Engl. J. Med.*, **265**, 253-267.

BAETJER, A.M. (1950). Pulmonary carcinoma in chromate workers I. A review of the literature and report of cases. *AMA Arch. Industr. Hyg. Occup. Med.*, **2**, 487-504.

BAETJER, A.M., DAMRON, C. & BUDACZ, V. (1959). The distribution and retention of chromium in men and animals. *AMA Arch. Industr. Hlth.*, **20**, 136-150.

BECHER, H., JEDRYCHOWSKI, W., WAHRENDORF, J., BASA-CIERPIALEK, Z., FLAK, E. & GOMOLA, K. (1993). Effects of occupational air pollutants on various histological types of lung cancer: a population based case-control study. *Br. J. Industr. Med.*, **50**, 136-142.

BIDSTRUP, P.L. (1951). Carcinoma of the lung in chromate workers. *Br. J. Industr. Med.*, **8**, 302-305.

DOLL, R. & PETO, R. (1978). Cigarette smoking and bronchial carcinoma: dose and time relationships among regular smokers and lifelong non-smokers. *J. Epidemiol. Community Hlth.*, **32**, 303-313.

DOLL, R., HILL, A.B., KREYBERG, L. (1957). The significance of cell type in relation to the aetiology of lung cancer. *Br. J. Cancer*, **11**, 43-48.

HAMMAR, S.P. (1994). Common neoplasms. In *Pulmonary Pathology*, 2nd edn. Dail, D.H. & Hammar, S.P. (eds) pp. 1123-1278. Springer: New York.

HUEPER, W.C. (1966). *Occupational and Environmental Cancers of the Respiratory System. Recent Results of Cancer Research, Vol. 3.* Springer: Berlin.

ISHIKAWA, Y., NAKAGAWA, K., SATOH, Y., KITAGAWA, T., SUGANO, H., HIRANO, T. & TSUCHIYA, E. (1994). 'Hot-spots' of chromium accumulation at the bifurcations of chromate workers' bronchi. *Cancer Res.*, **54**, 2342-2346.

KREYBERG, L. (1969). *Aetiology of Lung Cancer.* Universitetsforlaget: Oslo.

LANGARD, S. & NORSETH, T. (1975). A cohort study of bronchial carcinomas in workers producing chromate pigments. *Br. J. Industr. Med.*, **32**, 62-65.

LEHMANN, K.B. (1932). 1st Grund zu einer besonderen Beunruhigung wegen des Auftretens von Lungenkrebs bei Chromatarbeitern vorhanden? *Zentralblatt für Gewerbehygiene*, **19**, 168-170.

MACHLE, W. & GREGORIUS, F. (1948). Cancer of the respiratory system in the United States chromate-producing industry. *Publ. Hlth. Rep.*, **63**, 1114-1127.

MANCUSO, T.F. & HUEPER, W.C. (1951). Occupational cancer and other health hazards in a chromate plant: a medical appraisal. 1. Lung cancers in chromate workers. *Industr. Med. Surg.*, **20**, 358-363.

MIZUNO, S. & AKIBA, S. (1989). Smoking and lung cancer mortality in Japanese men: estimates for dose and duration of cigarette smoking based on the Japanese Vital Statistics data. *Jpn J. Cancer Res.*, **80**, 727-731.

MORITA, T. & SUGANO, H. (1990). A statistical analysis of lung cancer registered in the Annual of Pathological Autopsy Cases in Japan between 1958-1987, with special reference to the characteristics of lung cancer in Japan. *Acta Pathol. Jpn*, **40**, 665-675.

MURAO, M., OHSAKI, Y., ABE, S., KIMURA, K., TSUNETA, Y. & MIKAMI, H. (1981). *Surveillance Study on Occupational Diseases of Workers Engaged in Production of Chromate* (in Japanese). The First Department of Internal Medicine, Faculty of Medicine, University of Hokkaido: Sapporo.

NAKAGAWA, K., MATSUBARA, T., KINOSHITA, I., TSUCHIYA, E. SUGANO, H. & HIRANO, T. (1984). Surveillance study of a group of chromate workers - early detection and high incidence of lung cancer (in Japanese). *Lung Cancer*, **24**, 301-310.

NEWMAN, D. (1890). A case of adeno-carcinoma of the left inferior turbinate body and perforation of the nasal septum in the person of a worker in chrome pigments. *Glasgow Med. J.*, **33**, 469-470.

OHSAKI, Y., ABE, S., HOMMA, Y., YOZAWA, K., KISHI, F., MURAO, M., SATO, H., DATE, F., KAWAUCHI, F., KOBAYASHI, T. & FUJITA, I. (1974). High incidence of lung cancer in chromate workers (in Japanese). *J. Jpn Soc. Intern. Med.*, **63**, 1198-1203.

OHSAKI, Y., ABE, S., KIMURA, K., TSUNETA, Y., MIKAMI, H. & MURAO, M. (1978). Lung cancer in Japanese chromate workers. *Thorax*, **33**, 372-374.

PFEIL, E. (1935). Lungentumoren als Berufskrankung in Chromatbetrieben. *Deutsche Medizinische Wochenschrift*, **61**, 1197-1200.

SHIMOSATO, Y., KODAMA, T. & KAMEYA, T. (1982). Morphogenesis of peripheral type adenocarcinoma of the lung. In *Morphogenesis of Lung Cancer*, Vol. I, Shimosato, Y., Melamed, M.R. & Nettesheim, P. (eds) pp. 65-89. CRC Press: Boca Raton, FL.

SPENCER, H. (1985). *Pathology of the Lung*, 4th ed. Pergamon Press: Oxford.

STAYNER, L.T. & WEGMANN, D.H. (1983). Smoking, occupation and histopathology of lung cancer: a case-control study with the use of the Third National Cancer Survey. *J. Natl Cancer Inst.*, **70**, 421-425.

TSUCHIYA, E., KITAGAWA, T., OH, S., NAKAGAWA, K., MATSUBARA, T., KINOSHITA, I. & SUGANO, H. (1987). Incidence of squamous metaplasia in large bronchi of Japanese lungs: relation to pulmonary carcinomas of various subtypes. *Jpn J. Cancer Res.*, **78**, 559-564.

TSUNETA, Y., OHSAKI, Y., KIMURA, K., MIKAMI, H., ABE, S. & MURAO, M. (1980). Chromium content of lungs of chromate workers with lung cancer. *Thorax*, **35**, 294-297.

VALENTINE, E.H. (1957). Squamous metaplasia of the bronchus - a study of metaplastic changes occurring in the epithelium of the major bronchi in cancerous and noncancerous cases. *Cancer*, **10**, 272-279.

WAKIMOTO, J., TSUCHIYA, E., KITAGAWA, T., SATO, E., MATSUBARA, T., NAKAGAWA, K., KINOSHITA, I. & SUGANO, H. (1982). A histological analysis on squamous metaplasia of the bronchial epithelium in resected lung cancer cases (in Japanese). *Jpn J. Cancer Clin.*, **28**, 1709-1715.

WHO (1975). *Histological Typing of Female Genital Tract Tumours, International Histological Classification of Tumours*, Vol. 13. World Health Organization: Geneva.

WHO (1981). *Histological Typing of Lung Tumours*, 2nd edn, *International Histological Classification of Tumours*, Vol. 1. World Health Organization: Geneva.